# Cardiac metastasis from colon cancer effectively treated with 5-fluorouracil, leucovorin, and oxaliplatin (modified FOLFOX6) plus panitumumab: a case report

**DOI:** 10.1186/s12885-017-3147-2

**Published:** 2017-02-23

**Authors:** Yoshiki Tsujii, Yoshito Hayashi, Akira Maekawa, Tetsuji Fujinaga, Kengo Nagai, Shunsuke Yoshii, Akihiko Sakatani, Satoshi Hiyama, Shinichiro Shinzaki, Hideki Iijima, Tetsuo Takehara

**Affiliations:** Department of Gastroenterology and Hepatology, Osaka University Graduate School of Medicine, 2-2 Yamadaoka, Suita, Osaka 565-0871 Japan

**Keywords:** Cardiac metastasis, Colon cancer, Panitumumab, Anti-EGFR antibody, Molecular targeted agent

## Abstract

**Background:**

Cardiac metastasis from colorectal cancer is rare. There is little evidence supporting the effectiveness of chemotherapy, and standard therapy for metastatic cardiac tumors has not been established.

**Case presentation:**

A 76-year-old woman presented with a right ventricle tumor that was detected incidentally on screening cardiac ultrasonography. The initial computed tomography (CT) scan showed the cardiac tumor, which was approximately 40 mm in size, and multiple pulmonary nodules. Serum levels of tumor markers CEA and CA19-9 were elevated aberrantly. The suspected primary tumor, a well-differentiated adenocarcinoma of the transverse colon with wild-type *KRAS* was found by colonoscopy, and treatment with 5-fluorouracil, leucovorin, and oxaliplatin (modified FOLFOX6) plus panitumumab was initiated. After 4 courses of the therapy, a CT scan showed that the cardiac tumor size had markedly decreased and the pulmonary nodules had diminished. The serum levels of CEA and CA19-9 were also markedly decreased. After 12 courses of chemotherapy during 10 months of treatment, the patient continued to show a partial response, and she remained asymptomatic with continuation of the treatment through 15 courses.

**Conclusion:**

To the best of our knowledge, this is the first report of the efficacy of combination therapy using cytotoxic and molecular targeted agents against cardiac metastasis from colon cancer.

## Background

Colorectal cancer (CRC) is one of the three most commonly diagnosed malignancies worldwide, and it is a leading cause of death due to cancer [1]. The common sites of metastases from CRC are the lymph nodes, liver, and lungs. Autopsy studies have shown that the incidence of cardiac metastasis from CRC is 1.4 to 7.2% [2–4]. To date, however, only 12 cases of cardiac metastases from CRC have been reported in the English literature [5–7]. The prognosis for patients with metastatic cardiac tumors is poor, and chemotherapy alone against cardiac metastasis from CRC has rarely been effective [8]. Recently, anti-epidermal growth factor receptor (EGFR) monoclonal antibodies such as cetuximab or panitumumab have provided clinical benefit in patients with *KRAS* wild-type CRC [9, 10]. We herein present a case of cardiac metastasis from CRC that showed an appreciable response to combination therapy with oxaliplatin-based chemotherapy and panitumumab.

## Case presentation

**Fig. 1:**
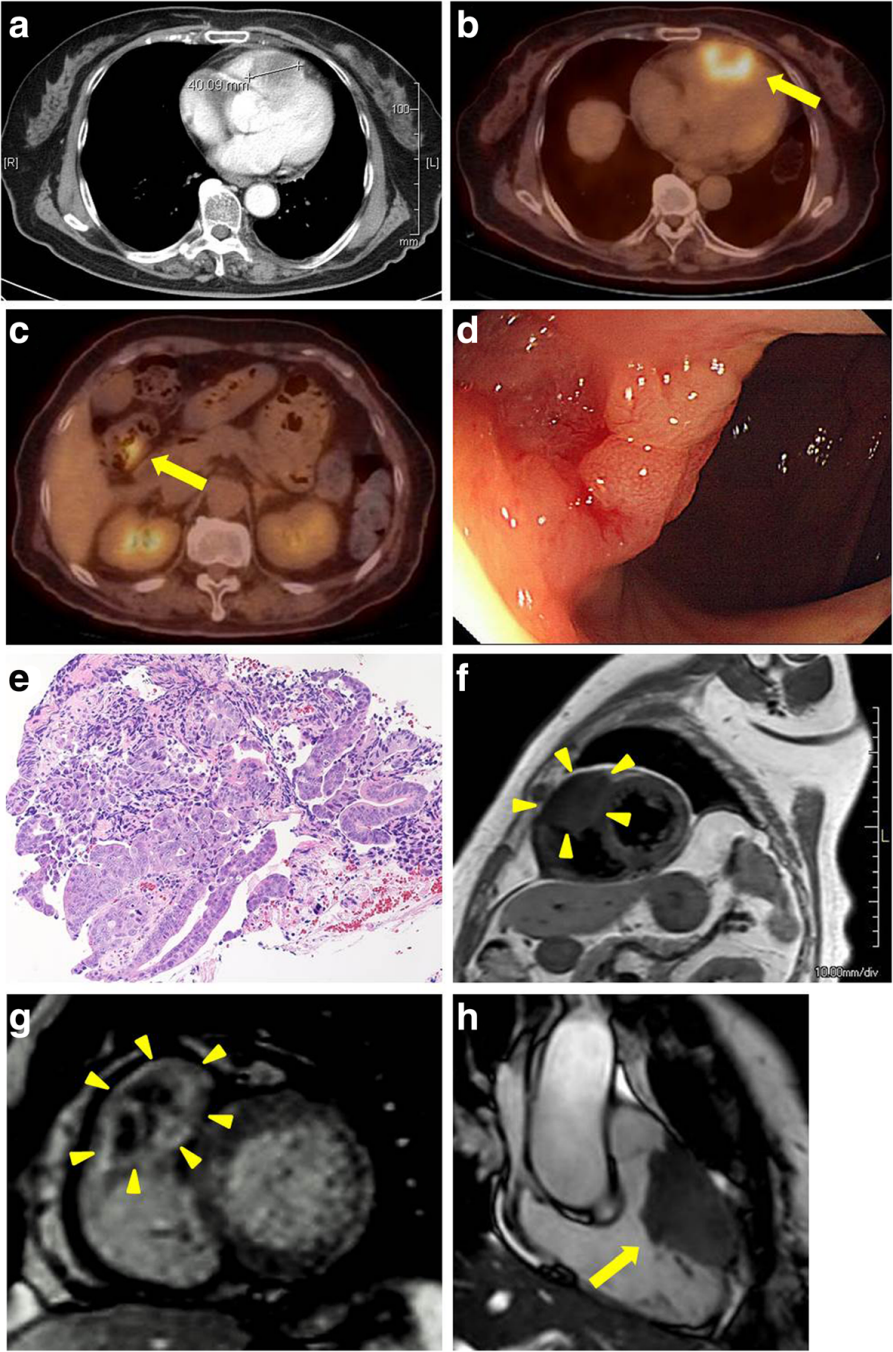
Initial diagnostic evaluation. Pre-treatment computed tomography (CT) scan showing the cardiac tumor (**a**). Positron emission tomography-computed tomography (PET-CT) scan showing abnormal uptake of fluorodeoxyglucose in the right ventricular mass (SUV max; 5.4) (**b**) and the transverse colon (SUV max; 4.4) (**c**). Endoscopic view of the primary lesion in the transverse colon (**d**) and the biopsy specimen showing well-differentiated adenocarcinoma (**e**). Cardiac magnetic resonance imaging (MRI; T1-weighted image, sagittal view) showing the irregular 54 × 32 mm mass, which had nearly equal intensity to the intact myocardium (**f**). The tumor is heterogeneous and characterized by a ring enhancement (**g**). MRI (long-axis view of the right ventricle) showing the tumor location lateral to the outflow tract of the right ventricle (**h**)

A 76-year-old woman who was incidentally diagnosed with a tumor of the right ventricle by screening cardiac ultrasonography was referred to our hospital for further examination. Chest computed tomography (CT) showed the cardiac tumor, which was approximately 40 mm in size (Fig. 1a), and multiple pulmonary nodules. The patient had elevated serum tumor markers (CEA, 724 ng/mL; and CA19-9, 54 U/mL), and positron emission tomography-computed tomography (PET-CT) showed abnormal uptake of fluorodeoxyglucose in the cardiac mass, the pulmonary nodules, and the transverse colon (Fig. 1b and c). Colonoscopy confirmed a 25-mm type 2 tumor in the transverse colon, which was diagnosed as a well-differentiated adenocarcinoma with wild-type *KRAS* on histopathological examination (Fig. 1d and e). With regard to the cardiac tumor, dynamic magnetic resonance imaging (MRI) showed an irregular 54- × 32- × 31-mm mass that was nearly isointense to the intact myocardium on both T1-weighted and T2-weighted images and was characterized by a ring enhancement (Fig. 1f and g). The tumor was lateral to the outflow tract of the right ventricle (Fig 1h), fixed to the endocardium, and infiltrated the myocardium. Tissue biopsy was considered unsafe because of the location of the tumor. There were no electrocardiographic abnormalities, and there was no sign of systolic or diastolic dysfunction on the echocardiogram. The left ventricular ejection fraction was 69%, and the Doppler studies showed normal blood flow that was unobstructed by the tumor. The tumor was deemed inoperable by cardiovascular surgeons because of the myocardial invasion. Thus, based on imaging study findings, we diagnosed the heart and lung lesions as cardiac and pulmonary metastases from the primary colon cancer (UICC cT2N0M1b Stage IVb).

**Fig. 2:**
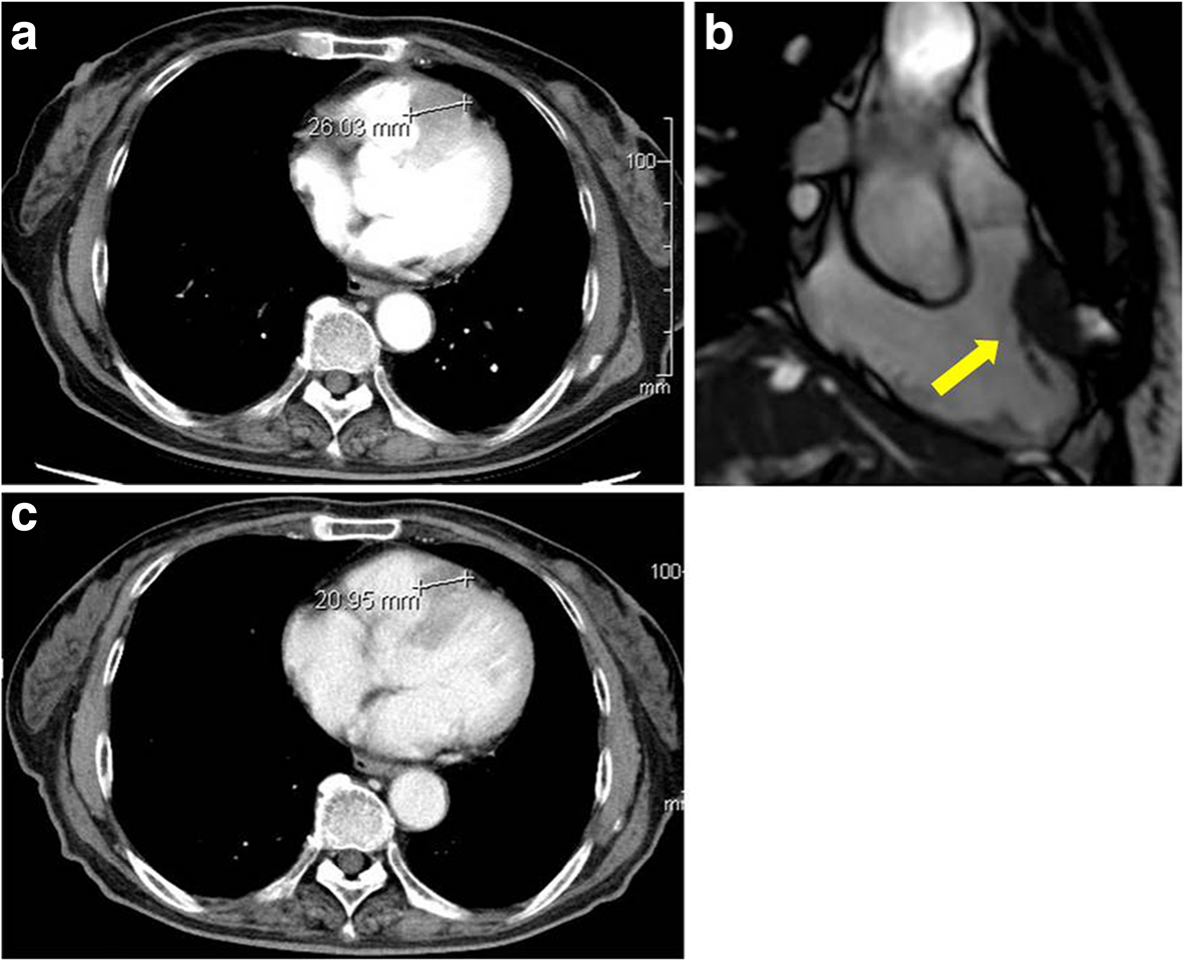
Therapeutic response. A follow-up computed tomography (CT) scan showing the smaller cardiac tumor after 3 months and 4 courses of chemotherapy] (**a**). A follow-up magnetic resonance imaging (MRI) after 8 courses of chemotherapy also shows a sustained reduction in the tumor size (**b**). CT after 10 months and 12 courses of chemotherapy (**c**)

The patient was treated with a modified 5-fluorouracil (400 mg/m^2^ bolus then 2400 mg/m^2^ 46-h infusion), leucovorin (200 mg/m^2^), and oxaliplatin (85 mg/m^2^) (mFOLFOX6) regimen plus panitumumab (6 mg/kg) every 2 to 3 weeks. A follow-up CT after 4 courses of chemotherapy showed that the cardiac tumor size decreased from 40 to 26 mm in size (Fig. 2a) and the multiple pulmonary nodules were also diminished. The serum CEA and CA19-9 levels were markedly decreased, from 724 to 29 ng/mL and 54 to 10 U/mL, respectively. The patient tolerated the treatment well. The most severe toxicity according to the common terminology criteria for adverse events (CTCAE, version 4.0) was grade 2 neuropathy that emerged after 6 courses of chemotherapy and was associated with the oxaliplatin. A follow-up MRI after 8 courses of chemotherapy indicated that the patient had sustained a prolonged significant response (Fig. 2b). Oxaliplatin was discontinued after 10 courses of chemotherapy due to neuropathy. The dosage of chemotherapy was also reduced (5-fluorouracil [320 mg/m^2^ bolus then 1920 mg/m^2^ 46-h infusion], leucovorin [200 mg/m^2^], and panitumumab [4.8 mg/kg] every 3 or 4 weeks). The patient remained asymptomatic with a prolonged partial response 10 months (12 courses of chemotherapy) after initial treatment (Fig. 2c). However, a follow-up CT after 15 courses of chemotherapy indicated progressive disease. The primary CRC remained undetectable by CT, but the cardiac tumor increased to 31 mm in size. Both the serum CEA and CA19-9 levels continued to increase. Despite our recommendation for further treatment by a second-line regimen, the patient elected to receive only best supportive care. Currently, the patient is alive over 2 years after the diagnosis of the cardiac metastasis.

## Discussion and conclusions

Cardiac metastasis from CRC is rare. A progressive metastatic mass of the heart occasionally causes acute heart failure or superior vena cava syndrome, resulting in sudden death [7, 11, 12]. Because the incidence of cardiac metastases was reported to be 1.4 to 7.2% in autopsy studies, echocardiography or cardiac MRI may not be necessary for all CRC patients. However, echocardiography should be performed if advanced CRC patients exhibit any sign of heart disease.

MRI is considered useful for the diagnosis of cardiac tumors [13]. It can accurately recognize the pericardium, the myocardial walls, and the cardiac chambers, thereby allowing for evaluation of infiltration or extension of the tumor. Additionally, reference to the known distinctive appearance is useful for the differential diagnosis [14]. For example, angiosarcoma, which is one of the most common subtypes of sarcomas, has marked signal heterogeneity on T1- and T2-weighed images with hyperintense foci corresponding to intratumoral hemorrhage [15], and the feature of ring enhancement of a lesion, which we observed in the present case has also been documented in previous cases of cardiac metastasis from CRC [5, 6]. Thus, this finding seems valuable in the diagnosis of a secondary malignant cardiac tumor.

Concerning the treatment of cardiac metastasis, several reports emphasize the role of surgery based on significant improvement in survival times [16], and surgical resection for liver and pulmonary metastasis is now generally accepted and performed as a potentially curative treatment [17, 18]. However, the potential survival benefit from cardiac surgery may be counterbalanced by perioperative morbidity and mortality. Therefore, indications for surgery in patients with metastatic cardiac tumors must be carefully considered, particularly for asymptomatic elderly patients or those with other metastatic lesions, such as in this case.

Recently, anti-EGFR monoclonal antibodies (cetuximab or panitumumab) have become widely used and have improved the prognosis for patients with wild-type *RAS* metastatic CRC. Oxaliplatin-based chemotherapy plus panitumumab showed higher response rates compared to oxaliplatin-based chemotherapy alone, especially in achieving early tumor shrinkage, which is associated with improved progression-free survival and overall survival [19]. In clinical practice, we routinely performed only *KRAS* mutational analysis because of a lack of health insurance coverage. However, an extended *RAS* analysis was considered essential for this case; therefore, we conducted an analysis of banked patient tumor specimens, which revealed that the tumor had wild-type *KRAS* exon 2 (codons 12, 13), exon 3 (codons 59, 61), exon 4 (codons 117, 146) and *NRAS* exon 2 (codons 12, 13), exon 3 (codons 59, 61), and exon 4 (codons 117, 146). During the past decade, molecular targeted therapy has provided a promising strategy for clinical oncology, and metastatic cardiac tumors originating from renal cell carcinomas have been successfully treated with tyrosine kinase inhibitors (sunitinib or pazopanib) [20, 21]. However, we believe that ours is the first report of significant reduction of a metastatic cardiac tumor from a CRC by the use of molecular targeted agents.

It must be noted that the possibility of a primary cardiac tumor in the present case cannot be denied completely because the diagnosis of metastatic CRC could not be confirmed by biopsy. We treated the cardiac tumor as metastasis from CRC for the following reasons. First, primary tumors of the heart are mostly myxomas. In our patient, the abnormal uptake of fluorodeoxyglucose on the CT-PET scan strongly indicated a malignancy. Next, primary malignant tumors of the heart, such as sarcomas, lymphoma, and mesothelioma, are generally less common than metastases [22], and primary cardiac tumors are known to have the most unfavorable prognosis, as they are rapidly-growing and extremely refractory to chemotherapy [23]. In our patient, the response to chemotherapy, i.e., the tumor shrinkage and the decrease in the serum levels of tumor markers, supported the diagnosis of a metastatic cardiac tumor and indicated that the combination of cytotoxic and molecular targeted chemotherapy may be a suitable treatment for select patients with asymptomatic cardiac CRC metastases.

In conclusion, we encountered a patient who had a cardiac metastasis from CRC that responded well to oxaliplatin-based chemotherapy plus panitumumab. This indicates that the combination of cytotoxic and molecular targeted chemotherapy may be suitable for select patients with asymptomatic metastatic cardiac tumors.

## Abbreviations

CRC: Colorectal cancer
CT: Computed tomography
EGFR: Epidermal growth factor receptor
MRI: Magnetic resonance imaging
PET-CT: Positron emission tomography-computed tomography
